# Reconfigurable anomalous reflectors with stretchable elastic substrates at 140 GHz band

**DOI:** 10.1515/nanoph-2022-0758

**Published:** 2023-05-10

**Authors:** Yuto Kato, Kazuma Yonemura, Kento Seki, Retsuku Kambara, Atsushi Sanada

**Affiliations:** Research Institute for Physical Measurement, National Institute of Advanced Industrial Science and Technology, Ibaraki, 305-8563, Japan; Graduate School of Engineering Science, Osaka University, Osaka, 560-8531, Japan; School of Engineering Science, Osaka University, Osaka, 560-8531, Japan

**Keywords:** 6G communication, anomalous reflectors, metasurfaces, millimeter-wave, reconfigurable intelligent surfaces, stretchable elastic substrates

## Abstract

We propose reconfigurable anomalous reflectors with stretchable elastic substrates. The proposed reflector dynamically controls the reflection direction by mechanically stretching the substrate to induce a physical change of the unit cell period. Owing to the simple and scalable tuning mechanism, the proposed approach is applicable in the millimeter-wave and terahertz bands for a wide reflection steering. To demonstrate the proposed approach, stretchable anomalous reflectors are designed at 140 GHz for normal incident waves. From full-wave simulations, we numerically confirm that highly efficient anomalous reflections with suppressed parasitic reflections in the undesired directions are achieved toward shallower angles as the substrate is stretched. We experimentally demonstrate that the proposed reflectors allow a dynamic control of the reflection direction with wide steering ranges of more than 20°. Moreover, we confirm that the measured efficiencies of the anomalous reflections hardly deteriorate when stretching and maintain practically acceptable performances of over 50 %. The proposed stretchable reflectors have a potential to be used for a reconfigurable intelligent surface (RIS) that realizes dynamic optimizations of the wireless environment in the 6G communication.

## 1 Introduction

In the sixth generation (6G) wireless communication, millimeter-waves above 100 GHz are supposed to be used to realize high-speed and large-capacity communication with performances far exceeding those of 5G [1–3]. Since the millimeter-waves are highly susceptible to blockages from physical obstacles such as buildings and trees, coverage holes in non-line-of-sight environment are severe problems that must be addressed to realize 6G [1, 2, 4, 5]. One of the promising solutions for mitigating the coverage holes is using metasurface-based anomalous reflectors that reflect incident waves toward specified directions that do not necessarily adhere to the law of reflection [4, 6], [7], [8], [9], [10]. Due to the highly dynamic nature of wireless environment in mobile communications, it would be beneficial that anomalous reflectors be reconfigurable and capable of dynamically controlling the reflection direction. Reconfigurable anomalous reflectors are one form of reconfigurable intelligent surfaces (RISs) [5, 11], [12], [13] that have a potential of turning a highly probabilistic wireless environment into a programmable and deterministic space and leads to so to called the smart radio environment.

Various tuning technologies have been studied for the realization of reconfigurable anomalous reflectors [14–20]. Reconfigurable anomalous reflectors using pin/varactor diodes [21–25] have been demonstrated. However, the operating frequencies of these reflectors are limited to the microwave bands below 30 GHz owing to the finite package sizes of the diodes. In the millimeter-wave bands, reconfigurable anomalous reflectors using ferroelectric materials whose permittivity can be varied by a DC bias voltage have been demonstrated at 60 GHz [26]. Liquid crystals also have a potential for a tuning technology operating in the millimeter-wave bands [27–29]. However, the reflectors based on these materials suffer from a narrow steering range; for instance, the measured reflection angle shift in ref. [26] is only 8°. Moreover, the conventional reconfigurable reflectors generally require a complex bias network to feed a bias signal to discrete elements for controlling local reflection phases. In order to realize reconfigurable anomalous reflectors above 100 GHz for the 6G applications, it is essential to have a simple tuning mechanism capable of a wide reflection steering in the millimeter-wave bands.

In this paper, we propose a tuning mechanism using stretchable elastic substrates for reconfigurable anomalous reflectors applicable in the millimeter-wave bands. Our stretchable reflector dynamically controls the reflection direction in a wide range by mechanically stretching the substrate to induce a physical change of the unit cell period, which is a simple and low-cost solution without a bias signal network. To demonstrate the proposed approach, stretchable anomalous reflectors are designed at 140 GHz. Here, the target frequency is selected considering the fact that the 140 GHz band is one of the most promising frequency bands for the 6G communication [1]. The reflection steering performances of the designed stretchable reflectors are investigated both numerically and experimentally. In this study, we realize stretchable reflectors by introducing a metallic strip array on the backside that can mimic a ground plane and retain the stretchability of the substrate, in contrast to conventional stretchable metasurfaces for transmission-type applications without a ground plane [30–33].

The remainder of this paper is organized as follows. In Section 2.1, we introduce the configuration of the stretchable anomalous reflector and present three designs at 140 GHz for normal incident waves with the nominal reflection angles of 45°, 60°, and 75° without a stretch. The reflection steering performances are numerically investigated in Section 2.2. Furthermore, the stretchable reflectors are fabricated, and the reflection steering performances are measured to evaluate the reflection steering ranges and efficiencies in Section 3. Finally, conclusions are presented in Section 4.

## 2 Reconfigurable anomalous reflectors with stretchable elastic substrates

### 2.1 Stretchable reflector design

**Figure 1:**
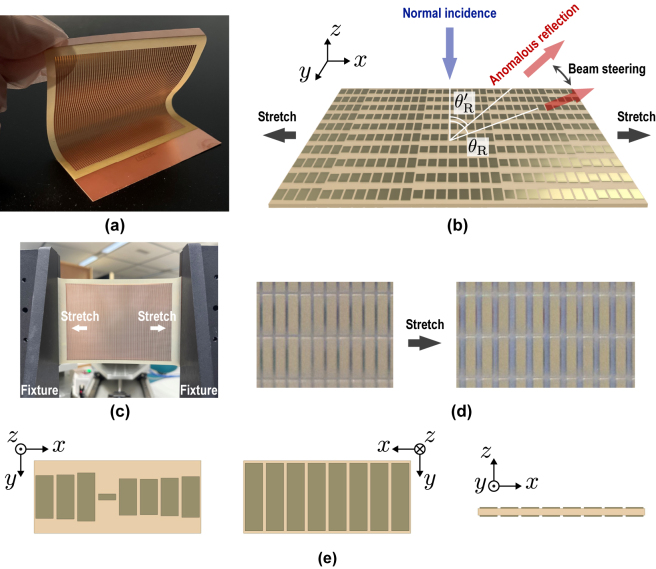
Reconfigurable anomalous reflector fabricated on a stretchable elastic substrate. (a) Photograph of a prototype. (b) Schematic of a dynamic control of the reflection direction. (c) Photograph of a stretched sample mounted on a fixture. (d) Photographs of the sample before and after a stretch (back side, *θ*_R0_ = 60°, approximately 130 % stretch ratio). (e) Front, back, and cross-sectional views of a typical unit cell (*θ*_R0_ = 60°).

The reconfigurable anomalous reflectors are designed with stretchable elastic substrates developed by Panasonic Industry (stretchable copper clad laminate called copper clad stretch [34]). The relative permittivity and loss tangent of the substrate at 10 GHz provided by the manufacturer are 2.3 and 0.025, respectively. Typical tensile strength and stretch ratio at break without metallization are 12.3 MPa and 227 %, respectively. The thickness of the substrate and those of the copper layers on both sides of the substrate are 100 µm and 18 µm, respectively. A photograph of a prototype is shown in Figure 1(a).

The dynamic control of the reflection direction with the proposed anomalous reflector is illustrated in Figure 1(b). Here, let the reflector be on the *xy*-plane in free space and illuminated with a *y*-polarized normal incident wave. The reflector consists of unit cells arranged periodically in the *x*- and *y*-directions with a reflection phase gradient in the *x*-direction. Let the periods of the unit cell be *D*_*x*_ and *D*_*y*_ in the *x*- and *y*-directions, respectively. The reflector reflects the normal incident wave toward the desired reflection direction with the reflection angle *θ*_R_. The anomalous reflection angle *θ*_R_ is changed by *D*_*x*_ according to the following equation [6]:

(1) $$\theta_{R}=\sin^{-1}\left(\lambda_{0}/D_{x}\right),$$

where *λ*_0_ is the wavelength in free space. Therefore, the reflection angle *θ*_R_ can be changed by a mechanical stretch of the elastic substrate with a physical change of the unit cell period *D*_*x*_ (see Figure 1(c) and (d)).

We design anomalous reflectors at 140 GHz for *θ*_R0_ = 45°, 60°, and 75° using a commercial electromagnetic simulator HFSS^®^, where *θ*_R0_ is the reflection angle without a stretch. Material parameter values used in the design are: the relative permittivity of 2.3, the loss tangent of 0.025, and the conductivity of 2.0 × 10^7^ S/m. Effect of material parameter inaccuracies due to their possible frequency characteristics on the reflection performances at 140 GHz will be discussed in Section 3.5. Figure 1(e) shows the unit cell of the reflector. It consists of 8 metallic strip elements on both sides of the substrate. The period *D*_*x*_ for the original state without stretching is determined by (1) as 3.04 mm, 2.48 mm, and 2.24 mm for the designs with *θ*_R0_ = 45°, 60°, and 75°, respectively, and *D*_*y*_ is chosen as *λ*_0_/2 = 1.07 mm. The lengths of the strips on the front side are optimized to efficiently reflect the normal incident wave toward the desired direction (*θ*_R0_) with suppressed parasitic reflections in the specular (0°) and symmetric (−*θ*_R0_) directions based on the nonlocal active-lossy design [6]. On the back side, metallic strips with a uniform length are arranged to maintain the stretchability of the substrate, instead of a solid ground metallic plane that prevents the substrate from stretching. By setting the strip length much longer than *λ*_g_/2, where *λ*_g_ is the guided wavelength along the substrate, the resonant frequency of the elements is sufficiently lowered from the design frequency and the strip array on the back side can mimic a ground plane. Please refer to the Supplementary Materials for the detailed dimensions of the unit cells.

### 2.2 Simulated reconfigurable anomalous reflection performances

Here, we present reconfigurable anomalous reflection performances of the designed reflectors evaluated by numerical simulations. In the simulations, each reflector is illuminated by a normal incident plane wave from the +*z*-direction at the Floquet port under the 2-D periodic boundary conditions in the *x*- and *y*-directions, and the reflections and transmissions into the spaces of *z* > 0 and *z* < 0, respectively, are calculated for each reflector.

**Figure 2:**
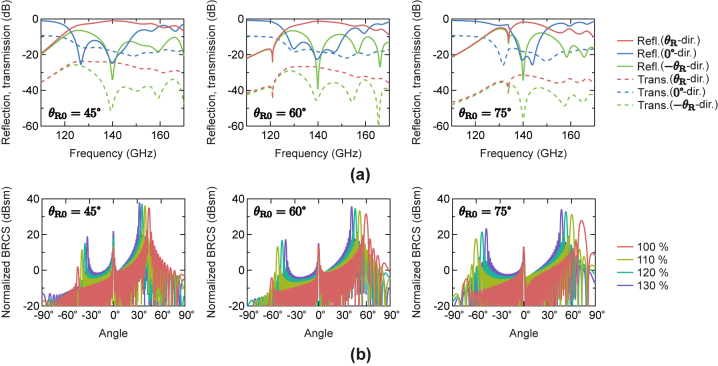
Simulated reconfigurable anomalous reflection characteristics. (a) Frequency dependences of S-parameters for designed reflectors without stretching. (b) Normalized bi-static radar cross sections (BRCSs) at 140 GHz for designed reflectors with the stretch ratios of 100 %, 110 %, 120 %, and 130 %.

First, the reflection characteristics for the designed reflectors with original *D*_*x*_ values without stretching are investigated. Figure 2(a) shows the simulated frequency dependences of the S-parameters for the propagating diffraction modes of each reflector design. It is noted that there are 3 propagating diffraction modes corresponding to the reflections/transmissions in the directions of *θ* = ±*θ*_R_ and 0°. As seen in the figure, most of the incident powers are reflected toward the desired *θ*_R_ direction with drastic parasitic reflection suppressions around 140 GHz. At 140 GHz, the reflection levels in the *θ*_R_ = *θ*_R0_ direction are −1.1 dB (78.4 %), −1.3 dB (74.7 %), and −1.8 dB (66.5 %) with the total parasitic reflections of −24.3 dB (0.4 %), −22.4 dB (0.6 %), and −22.6 dB (0.6 %) for the designs with *θ*_R0_ = 45°, 60°, and 75°, respectively. It is also seen from Figure 2(a) that the transmission powers passing to the back side of the reflectors are relatively low with the total parasitic transmissions at 140 GHz of −17.1 dB (1.9 %), −17.8 dB (1.6 %), and −18.1 dB (1.6 %) for the designs with *θ*_R0_ = 45°, 60°, and 75°, respectively. It is noted that the anomalous reflection angle *θ*_R_ is supposed to decrease with the frequency *f* = *c*_0_/*λ*_0_ according to (1), where *c*_0_ is the speed of light.

Then, the reflection steering performances of each reflector with stretching the substrate are evaluated by numerical simulations. In the simulations, it is assumed that the material parameters and the thickness remain constant in stretching the substrate. The reflection scattering performance of a target object is expressed by a bi-static radar cross section (BRCS) that represents the effective cross-sectional area of the object quantified by the scattering power toward a certain reflection angle [35]. Figure 2(b) shows the simulated BRCSs calculated for the stretched reflectors consisting of 30-unit cells periodically arranged in the *x*-direction. Considering practical use cases, we study the performances for the stretch ratios of 100 %, 110 %, 120 %, and 130 %. As seen from Figure 2(b), the reflection angle shifts shallower toward the normal direction as the substrate is stretched. The angle shift ranges are 12°, 18°, and 25° for the designs with *θ*_R0_ = 45°, 60°, and 75°, respectively, in stretching the substrate up to 130 % from the original state (100 %), which are consistent to the theoretical predictions from (1). Although the parasitic reflections in both specular (0°) and symmetric (−*θ*_R0_) directions are slightly increased as the substrate is stretched, they are still kept low even at 130 % stretch ratio; the parasitic reflections are lower than the reflection peaks by 16.1 dB, 18.4 dB, and 10.7 dB for the designs with *θ*_R0_ = 45°, 60°, and 75°, respectively. With these results, the dynamic control of the reflection direction with suppressed parasitic reflections using the stretchable anomalous reflectors is numerically demonstrated.

Table 1 presents the simulated reflection efficiencies and loss contributions owing to total parasitic reflections, total parasitic transmissions, and total material losses dissipated as the dielectric and conductor losses in the substrate for each reflector design at stretch ratios up to 130 %. It is seen from Table 1 that there is little deterioration or slight improvement in the efficiencies for the designs with *θ*_R0_ = 45° and *θ*_R0_ = 60° and 75°, respectively, when stretching. This can be attributed to the fact that the slight increases in the parasitic reflections when stretching are compensated by the decreases in the material losses. The reduction in material losses as the substrate is stretched with the reflection angle shifted to the shallower side is consistent with [10] in which the material losses of static anomalous reflectors increase with the reflection angle. The parasitic transmissions are kept low for the cases of stretch ratios up to 130 %. Note that these results of maintaining high efficiency anomalous reflections even when the substrates are stretched are not trivial, since the reflector designs are optimized only for structures without stretching.

**Table 1:** Efficiencies and loss contributions owing to parasitic reflections, parasitic transmissions, and material losses for designed reflectors with the stretch ratios of 100 %, 110 %, 120 %, and 130 %. Note that the sum of the efficiency and three loss contributions does not add up to 100 % in some cases due to the rounding of each number.

Stretch ratio	*θ*_R0_ = 45^o^				*θ*_R0_ = 60^o^				*θ*_R0_ = 75^o^			
	Efficiency	Parasitic	Parasitic	Material	Efficiency	Parasitic	Parasitic	Material	Efficiency	Parasitic	Parasitic	Material
		refl.	trans.	loss		refl.	trans.	loss		refl.	trans.	loss
100 %	78.4 %	0.4 %	1.9 %	19.2 %	74.7 %	0.6 %	1.6 %	23.1 %	66.5 %	0.6 %	1.6 %	31.4 %
110 %	78.9 %	0.6 %	2.1 %	18.4 %	76.6 %	1.1 %	2.0 %	20.3 %	71.7 %	3.1 %	1.8 %	23.4 %
120 %	78.3 %	1.4 %	2.3 %	18.0 %	77.2 %	1.5 %	2.0 %	19.3 %	71.5 %	5.3 %	1.8 %	21.4 %
130 %	77.3 %	2.6 %	2.2 %	17.9 %	77.5 %	1.6 %	2.0 %	18.9 %	71.2 %	6.5 %	1.7 %	20.6 %

## 3 Experiments

### 3.1 Prototypes

The designed reflectors for *θ*_R0_ = 60° and 75° are fabricated to experimentally demonstrate the reconfigurable anomalous reflection characteristics. By using standard printed circuit board lithography techniques, the prototypes are fabricated on the 0.1 mm-thick elastic stretchable substrates with 18 µm-copper metallic patterns on both sides. The metallic pattern area of each prototype is 70 mm × 70 mm with the total sample size of 80 mm × 100 mm. In order to hold the prototypes stably with a stretch fixture, metallic layers on the edges are left on both sides of the substrates as shown in Figure 1(a).

### 3.2 Measurement system

**Figure 3:**
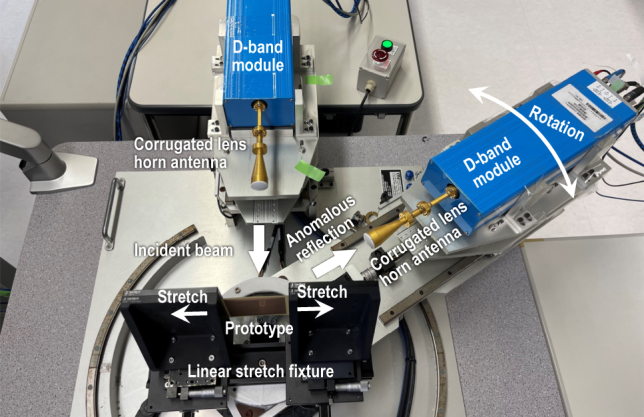
Photograph of the experimental system for measuring the reflection characteristics.

A photograph of the measurement system is shown in Figure 3. The stretched sample is set in a sample fixture with a linear stretching functionality and illuminated with a vertically polarized normal incident wave by a fixed transmitting antenna. By rotating a receiving antenna around the sample with an automated rotation stage, the angle *θ* dependencies of the reflection characteristics are measured for each prototype. The scanning range of *θ* is limited to −90° ≤ *θ* ≤ −40° and 40° ≤ *θ* ≤ 90° due to the collision of transmitting and receiving modules. Two identical corrugated lens horn antennas with a 28 dBi are used in the system.

For the specular reflection (*θ* = 0°), the direct reflection received by the transmitting antenna is measured. The full-width at half-maximum (FWHM) of the Gaussian beam from the antennas at the sample position is approximately 13 mm. Since the sample size is much larger than the beam width, the scattering from the sample fixture is considered to be negligible. The distance between each antenna and the sample center is set to be 150 mm. Please refer to ref. [10] for more information of the system.

### 3.3 Stretch fixture and the sample stretch ratio

As shown in Figure 1(c), both ends of the sample are held by the fixture and the sample is stretched symmetrically along the linear stage with micrometers so that the sample center remains on the axis of the rotation stage. The sample fixture distance for the original position without stretching is set to be 80 mm. The reflection characteristics are measured with the sample stretched with the fixture distance ranging from 100 % (80 mm) to 140 % (112 mm) in 10 % increments. In the following, we refer the stretch conditions of 100 %, 110 %, 120 %, 130 %, and 140 % as the conditions A, B, C, D, and E, respectively. It is noted that the sample stretch ratio differs from the fixture distance change ratio because the stress concentrates in gaps between the metallic patches on the elastic substrate. Thus, the relationship between the sample stretch ratio and the fixture distance is measured in the five stretch conditions by taking photographs of the surface metallic patterns along with a micro ruler in advance of the reflection measurements. The results are summarized in Table 2.

**Table 2:** Sample stretch ratios in the five experimental conditions.

Fixture distance change ratio	A	B	C	D	E
	100 %	110 %	120 %	130 %	140 %
Sample stretch ratio *θ*_R0_ = 60°	97.3 %	104.8 %	111.8 %	119.4 %	127.4 %
Sample stretch ratio *θ*_R0_ = 75°	98.8 %	106.4 %	114.2 %	120.7 %	128.9 %

### 3.4 Measured anomalous reflection characteristics without stretching

**Figure 4:**
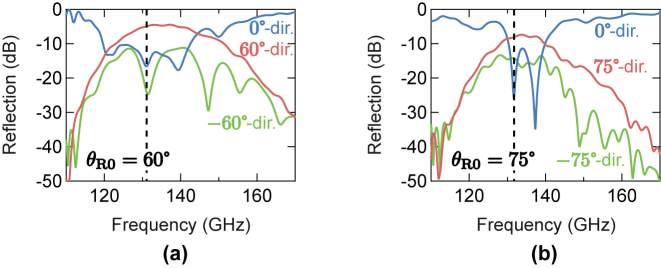
Measured frequency characteristics of the reflections in the three particular directions of *θ* = ±*θ*_R0_ and 0°. (a) Prototype with *θ*_R0_ = 60°. (b) Prototype with *θ*_R0_ = 75°.

First, we present the measured anomalous reflection characteristics for the prototypes without stretching. Figure 4 shows the measured frequency characteristics of the reflections in the three particular directions of *θ* = ±*θ*_R0_ and 0°. The reflection to *θ* = +*θ*_R0_ corresponds to the desired anomalous reflection, whereas those to *θ* = −*θ*_R0_ and 0° correspond to the parasitic reflections in the symmetric and specular directions, respectively. As seen in Figure 4, there are frequency bands with high desired anomalous reflections and suppressed parasitic reflections around 140 GHz. The dashed lines indicate the operating frequencies at which the parasitic reflections are locally minimized. These frequencies are 131.08 GHz and 131.76 GHz for *θ*_R0_ = 60° and 75°, respectively. Incidentally, for the prototype with *θ*_R0_ = 75°, the operating frequency is chosen at the first dip among the two in the specular reflection based on the comparison with the simulation results of Figure 2(a). At these operating frequencies, the reflection levels in the designed *θ*_R0_ directions are −4.9 dB and −8.1 dB with the total parasitic reflections of −15.8 dB and −13.9 dB for the prototypes with *θ*_R0_ = 60° and 75°, respectively. The frequency shifts from the designed frequency of 140 GHz are considered to be due to prototyping errors in fabrications and/or the inaccurate material parameter values used in the design.

### 3.5 Measured reflection steering performances

**Figure 5:**
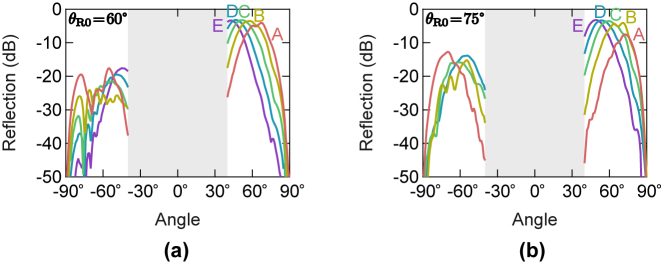
Measured angle dependencies of the reflections of the stretched reflectors in the five stretch conditions. (a) The results for the prototype with *θ*_R0_ = 60° measured at 131.08 GHz. (b) The results for the prototype with *θ*_R0_ = 75° measured at 131.76 GHz. The reflections in −40° ≤ *θ* ≤ 40° are not measured to avoid the collision of the transmitting and receiving modules.

Figure 5 shows the angle dependencies of the reflections of the reflectors when the substrate is stretched under the five conditions shown in Table 2. The results at the operating frequencies of 131.08 GHz and 131.76 GHz are shown for the prototypes with *θ*_R0_ = 60° and 75°, respectively. The reflections in −40° ≤ *θ* ≤ 40° are not measured to avoid the collision of the transmitting and receiving modules. It is seen from Figure 5 that the reflection angle shifts shallower as the substrate is stretched. The angle shift ranges are 24° and 23° for the prototypes with *θ*_R0_ = 60° and 75°, respectively, in stretching the substrate from the conditions A to E. It is noted that the measured angle shift ranges are much wider than the reported value of 8° for the ferroelectric material-based reflector at 60 GHz [26]. It is also noted that the parasitic reflections in the −*θ*_R_ directions remain low levels below −17.7 dB and −13.9 dB for the prototypes with *θ*_R0_ = 60° and 75°, respectively, even with the stretch. As shown in the Supplementary Materials, the reflections in the specular direction are also suppressed during the stretch and remain below −9.3 dB and −11.6 dB for *θ*_R0_ = 60° and 75°, respectively. Incidentally, it is also shown in the Supplementary Materials that the reproducibility of the reflection characteristics is confirmed when returning to the original state after the stretch. In addition, the prototype with *θ*_R0_ = 75° has been broken during the stretch in the condition E, thus the reflections in −90° ≤ *θ* ≤ −40° for *θ*_R0_ = 75° under the stretch condition E are not measured. Note that the stretch ratio at break of a stretchable reflector made of the elastic substrate is much smaller than that of the bare elastic substrate of 227 % due to the stress concentration in gaps between metallic patches. Improving the durability of stretchable reflectors is important for some applications. To this end, in addition to efforts from the material development side to increase the strength of stretchable elastic substrates, detailed stress analysis in a reflector for optimizing a structure with alleviated stress concentration would be required.

**Figure 6:**
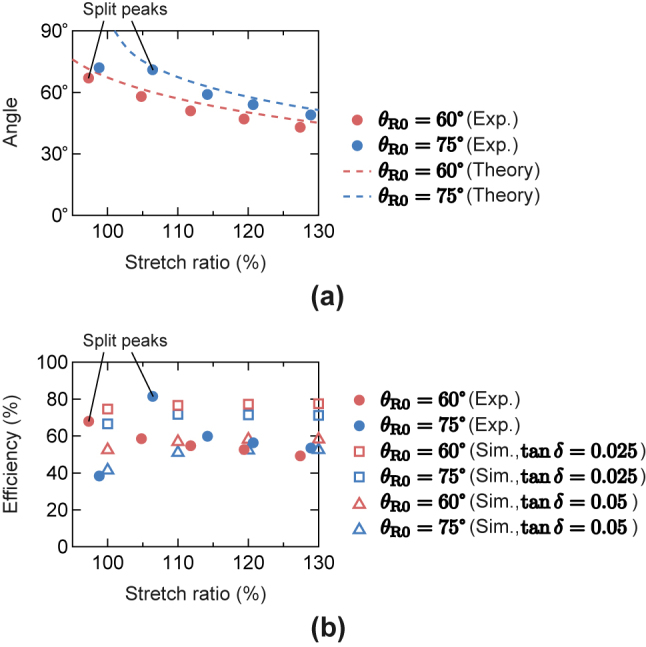
Measured reflection steering performances for the prototypes with *θ*_R0_ = 60° and 75° (at 131.08 GHz and 131.76 GHz, respectively). (a) Reflection angles. (b) Reflection efficiencies.

Figure 6(a) shows the reflection angles plotted against the sample stretch ratio obtained from the measured reflection characteristics in Figure 5. In Figure 6(a), the theoretical predictions from (1) are also shown as dashed lines. As seen in Figure 6(a), the angle shift ranges of the measurement results agree well with the theoretical predictions, except for that of the prototype with *θ*_R0_ = 75° under the condition A. However, the measured results are shifted to shallower angles than the calculated results, especially for that of the prototype with *θ*_R0_ = 75° under the condition A. This discrepancy can be attributed to the effect of the narrow incident beam width, which becomes apparent for a deep anomalous reflection angle [10], and does not directly reflect the reflection characteristics of the prototypes in an actual use environment where a much larger reflector is illuminated from a distant base station antenna with a plane wave. The in-plane non-uniformity of the stretching may also partially affect deviated reflection angles from the theoretical predictions. It is also noted that the measurement results of the prototypes with *θ*_R0_ = 60° and 75° under the stretch conditions A and B, respectively, are obtained from split resonant peaks as shown in Figure 5.

The reflection efficiencies of the stretched prototypes are estimated from the measured reflection peaks in Figure 5 based on the physical optics approximation that considers the effect of the focused beam [6, 10]. The obtained efficiencies of the two prototypes are shown in Figure 6(b) as filled circle markers. As seen in Figure 6(b), the efficiencies do not deteriorate when the substrates are stretched and maintain practically acceptable performances of over 50 %, which is qualitatively consistent with the simulation results of Table 1. However, the measured efficiencies are lower than the simulation results using the nominal loss tangent value of 0.025 at 10 GHz provided from the manufacturer (shown with unfilled square markers in Figure 6(b)). This discrepancy can be explained by the increase in the loss tangent at 140 GHz from the nominal value. According to additional simulations with variable loss tangent values, the measured results agree well with the simulation results using the doubled loss tangent of 0.05 (shown with unfilled triangle markers in Figure 6(b)) except for the results obtained from split resonances. With these results, dynamic control of the reflection direction with wide steering range and acceptable efficiency is experimentally demonstrated using the reconfigurable anomalous reflectors made on the elastic stretchable substrates.

## 4 Conclusions

We have proposed the reconfigurable anomalous reflectors with stretchable elastic substrates and demonstrated their reflection steering performances at 140 GHz for the 6G applications. The proposed reflector dynamically controls the reflection direction by mechanically stretching the substrate to induce a physical change of the unit cell period, which is a simple and low-cost solution for an RIS. It has been numerically confirmed that highly efficient anomalous reflections with suppressed parasitic reflections are achieved toward shallower angles as the substrate is stretched, in spite of the reflector designs optimized only for unstretched structures. The designed reflectors have been fabricated on the elastic substrates. We have successfully demonstrated the dynamic control of the reflection direction with the measured angle shifts of 24° and 23° for the prototypes with *θ*_R0_ = 60° and 75°, respectively, by stretching the substrate up to approximately 130 % stretch ratio. The measured efficiencies hardly deteriorate when stretching and maintain practically acceptable performances of over 50 % with suppressed parasitic reflections in the undesired directions. Owing to the simple and low cost configuration, the proposed stretchable reflectors have a potential to be used for dynamic optimizations of the wireless environment in the 6G communication. In addition, the concept is fundamentally scalable and potentially applicable to much higher terahertz frequency regions for various dynamic photonic applications such as a light emitter and sensor. One drawback of the stretchable reflectors is a slow response speed compared to the electrically controlled ones, which suggests that the two types of reconfigurable reflectors are suitable for different applications. The stretchable reflectors are suitable for applications where a relatively slow response speed of second- to millisecond-order is sufficient such as a user-tracking system for the smart radio environment. Future research includes shifting the reflection angle in both the azimuth and elevation axes by introducing phase gradients and stretching the substrate in both the *x*- and *y*-directions, realizing anomalous reflectors operating with both polarizations, and extending the operating frequency up to 300 GHz.

## Supplementary Material

Supplementary Material Details
