# Supplementary material for: Reconfigurable anomalous reflectors with stretchable elastic substrates at 140 GHz band
Source: Nanophotonics. 2023 May 10;12(13):2527–35. doi: 10.1515/nanoph-2022-0758 (PMC11501655; doi:10.1515/nanoph-2022-0758)
Supplement: Supplementary file 1 — Supplementary Material Details [file j_nanoph-2022-0758_suppl_001.pdf]

Supplementary Materials for

Reconfigurable Anomalous Reflectors with Stretchable Elastic Substrates at 140 GHz Band

Yuto Kato[1*], Kazuma Yonemura[2], Kento Seki[2], Retsuku Kambara[3], and Atsushi Sanada[2]

[1]National Institute of Advanced Industrial Science and Technology, Research Institute for Physical Measurement, Ibaraki, 305-8563, Japan

[2]Osaka University, Graduate School of Engineering Science, Osaka, 560-8531, Japan

[3]Osaka University, School of Engineering Science, Osaka, 560-8531, Japan

[*]Corresponding author: Yuto Kato (Email: y-katou@aist.go.jp)

S1 Unit cell dimensions of the designed reflectors

The metallic patterns of the front and back sides of the unit cells for the designed reflectors with $\theta_{\mathrm{R0}} = 45°$, 60°, and 75° are shown in Figs. S1(a)-(c). The widths of each metallic strip on both sides are set to be 0.33 mm, 0.26 mm, and 0.23 mm for the designs with $\theta_{\mathrm{R0}} = 45°$, 60°, and 75°, respectively. The gaps between adjacent strips in the x-direction on both sides are set to be 0.05 mm for all the designs. The strip lengths of 8 metallic patches on the front side of each unit cell are set as shown in Fig. S1(d), whereas the strip lengths on the back side are uniformly set to be 1 mm. The unit cell periods are given in the main text.

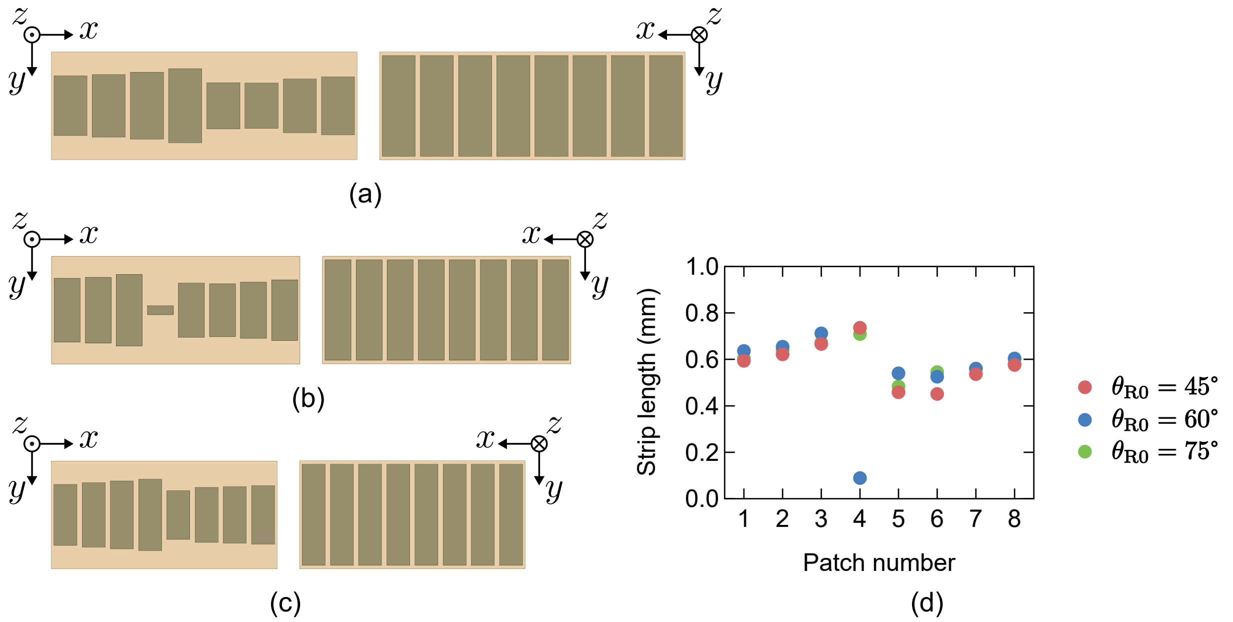

Fig. S1 Unit cells of the designed reflectors with (a) $\theta_{\mathrm{R0}} = 45°$, (b) 60°, and (c) 75°. (d) Strip lengths on the front side of each unit cell.

S2 Measured specular reflections of the reflectors with stretching the substrate

Fig. S2 shows the measured frequency characteristics of the specular reflections of the reflectors when the substrate is stretched under the five experimental conditions A–E. The dashed lines indicate the operating frequencies of 131.08 GHz and 131.76 GHz for $\theta_{\mathrm{R0}} = 60°$ and 75°, respectively. As seen in Fig. S2, the specular

reflections are kept relatively low at each operating frequency when the substrate is stretched, remaining below -9.3 dB and -11.6 dB for $\theta_{\mathrm{R0}} = 60°$ and $75°$, respectively.

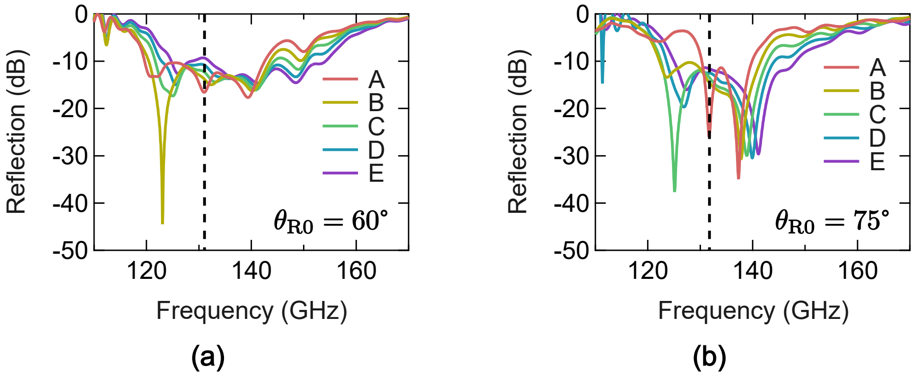

Fig. S2 Measured specular reflections of the reflectors when the substrate is stretched under the five experimental conditions A–E. (a) Prototype with $\theta_{\mathrm{R0}} = 60°$. (b) Prototype with $\theta_{\mathrm{R0}} = 75°$.

S3 Reproducibility of the reflection characteristics of the stretchable reflector

For the prototype with $\theta_{\mathrm{R0}} = 75°$, the reproducibility of the reflection characteristics is checked when returning to the original state after a stretch of approximately 125 % sample stretch ratio before the measurements of Section 3.3 and 3.4 of the main text. Fig. S3 shows the measured frequency characteristics of the reflections in the three particular directions of $\theta = \pm 75°$ and $0°$. The solid and dashed lines in Fig. S3 correspond to the reflections before and after the stretch, respectively. It is seen from Fig. S3 that the reproducibility is clearly confirmed not only for the anomalous reflection ($75°$) but also for the parasitic reflections in the symmetric ($-75°$) and specular ($0°$) directions.

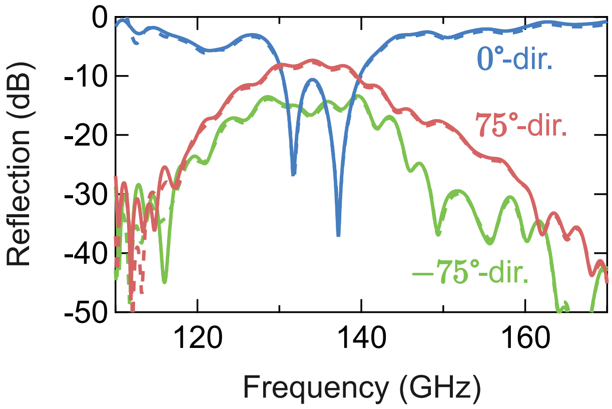

Fig. S3 Measured frequency characteristics of the reflections in the three particular directions of $\theta = \pm 75°$ and $0°$ for the prototype with $\theta_{\mathrm{R0}} = 75°$ before (solid lines) and after (dashed lines) the stretch.
